# Laboratory Surveillance, Quality Management, and Its Role in Addressing Antimicrobial Resistance in Africa: A Narrative Review

**DOI:** 10.3390/antibiotics12081313

**Published:** 2023-08-14

**Authors:** Khalid Musa, Ijeoma Okoliegbe, Tassabeeh Abdalaziz, Ahmed Taha Aboushady, John Stelling, Ian M. Gould

**Affiliations:** 1 Department of Medical Microbiology and Virology, Aberdeen Royal Infirmary, Aberdeen AB25 2ZN, UK; ijeoma.okoliegbe@nhs.scot (I.O.); tassabeeh.abdalaziz@nhs.scot (T.A.); ian.gould@nhs.scot (I.M.G.); 2 Department of Acute Medicine and Infectious Diseases, Aberdeen Royal Infirmary, Aberdeen AB25 2ZN, UK; 3 Division of Infectious Diseases, Brigham and Women’s Hospital, Harvard Medical School, Boston, MA 02115, USA; aaboushady@whonet.org (A.T.A.); jstelling@whonet.org (J.S.)

**Keywords:** antimicrobial resistance, AMR, antimicrobial resistance in Africa, quality management, AMR surveillance

## Abstract

AMR is a major public health concern that calls for extensive work and a multidisciplinary team approach. The high prevalence of infectious diseases in African nations leads to widespread antibiotic usage and eventual antimicrobial resistance, which has significant negative effects on people’s health, the economy, and society. Additionally, inadequate or nonexistent antimicrobial drug regulations, inappropriate prescription practices, and restrictions on public health prevention initiatives such as immunization, water and sanitation, and sexual health may all contribute to the emergence of AMR. Despite the need for laboratory quality assurance, many African laboratories confront substantial difficulties in implementing efficient quality assurance programs. AMR surveillance in Africa is hampered by a lack of laboratory capacity, insufficient data collection and analysis, and poor stakeholder collaboration. Several initiatives and programs, including the World Health Organization’s Global Antimicrobial Resistance and Use Surveillance System (GLASS), the Africa Centres for Disease Control and Prevention (Africa CDC) Antimicrobial Resistance Surveillance Network (AMRSNET), and the Fleming Fund, a UK government initiative aimed at tackling AMR in low- and middle-income countries, have been established to strengthen AMR surveillance in Africa and globally.

## 1. Introduction

Antibiotics serve a key role in lowering the global burden of communicable illnesses. However, the ability of pharmaceuticals to cure infectious diseases and the efficacy of drugs are not limitless. AMR jeopardizes the efficacy of infection treatment and is a public health issue with national and global implications. The spreading AMR pandemic is a global public health problem with far-reaching health, economic, and societal consequences [1]. Resistance develops as a result of selection pressure from rational and indiscriminate antimicrobial usage in human health, veterinary medicine, agriculture, and the environment. Infections produced by resistant bacteria result in prolonged illness duration, greater fatality rates, and increased expenses associated with alternative treatment, and AMR is seen as a global economic threat [2].

One of the biggest concerns to public health in the twenty-first century is AMR. It is critical to have knowledge about the top pathogen-drug combinations contributing to the burden of bacterial AMR, trends around the world, and the current size of the burden. If AMR continues to grow, many infections could become far more deadly than they are today [3].

Among African nations, communicable diseases continue to be the largest cause of death, accounting for the great majority of fatalities among children under the age of five. In comparison to other World Health Organization (WHO) areas, Africa has a much greater level of poverty and communicable disease risk factors, which are highly correlated with the number of infectious illness cases recorded [4].

AMR is a significant worldwide health problem that necessitates extensive work and a diverse team approach. Tracking AMR trends, patterns, treatments, and policy responses can be performed with the help of surveillance. The formulation of logical actions and policies is made easier by the proper correlation of surveillance data, which provide insightful information about the underlying epidemiology [5]. This paper’s goal is to increase the awareness of AMR surveillance in Africa and to highlight the existing situation of AMR in Africa.

## 2. Antimicrobial Resistance in Africa

The high prevalence of infectious diseases in African nations leads to widespread antibiotic usage and eventual antimicrobial resistance, which has significant negative effects on people’s health, the economy, and society. The lack of availability or access to antimicrobial treatments, as well as the possibility of low-quality or counterfeit medications when they are accessible, are the key factors aggravating the AMR problem in Africa. Additionally, antimicrobials, including antibiotics, can be purchased over the counter or fed to animals as preventative medicine or growth promoters in low-resource environments such as Africa [4].

Despite the fact that antimicrobial resistance emerges and spreads naturally, there is a higher selection pressure that is primarily brought on by the overuse and abuse of antibiotics in both human and animal medicine. In developing nations where strict rules on antibiotic use are not entirely in place and implemented, livestock production is rising. Large quantities of antibiotics, such as penicillin, β-lactams, fluoroquinolones, and aminoglycosides, are used by farmers on their livestock. Additionally, sub-therapeutic levels are used in animal feeds to promote productivity, reduce illness, and promote growth. High rates of resistance to the main antimicrobials used in human treatments in bacteria isolated from food-producing animals have been linked to the abuse of antibiotics. Bacteria isolated from plants and the environment have high reported rates of resistance [6].

The majority of low-resource settings still deal with outdated facilities, a lack of technical expertise, and a shortage of essential materials for AMR diagnosis and treatment. In low- and middle-income (LMIC) nations, this results in an increase in infectious diseases and AMR. Even in some national reference laboratories, AMR detection in LMICs is poor, which is indicative of a lack of laboratory capacity to support diagnostic processes [7].

The WHO announced a priority list for discovering novel and effective antibiotic therapies in 2017 [8]. The list was created to help guide research and development priorities for new antibiotics, with a focus on bacteria with multidrug resistance that cause serious and frequently fatal infections in healthcare and nursing home settings. Although the goal of this list was to establish new antibiotic studies and development priorities instead of identifying the most challenging pathogen–drug combinations, its utility in determining priorities has been limited by the lack of an ongoing nationwide evaluation concerning the burden of bacterial AMR [8].

In 2018, the African Union (AU) formed a Task Force on Antimicrobial Resistance in conformity with the United Nations (UN) strategy and to fulfil the specific needs of African nations. This Task Force represents AU agencies working in the human, animal, and plant health sectors to measure, prevent, and minimize the effects caused by AMR bacteria [9].

**Figure 1:**
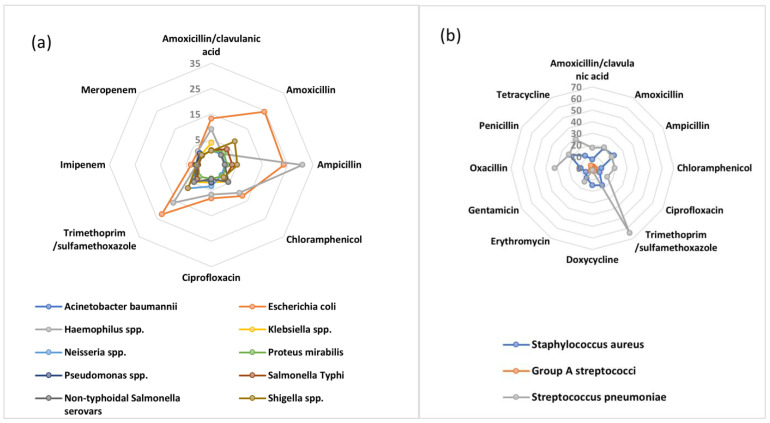
AMR patterns among (**a**) Gram−negative and (**b**) Gram−positive bacteria in the WHO African Region, 2016–2020 to first- and second-line antibiotics.

AMR typically occurs in microorganisms that cause pneumonia, diarrheal illnesses, tuberculosis (TB), sexually transmitted diseases, and malaria in Africa. These pathogens are among those that are likely to be spread within the population. Fighting TB, malaria, pediatric dysentery, and pneumonia now costs much more money due to drug resistance. Additionally, it jeopardizes efforts to successfully treat HIV/AIDS patients [10]. In a recent attempt to define the AMR burden in Africa, the WHO African region carried out a systematic review comprising isolates from 2016 to 2020. They concluded that *Escherichia coli* (*E. coli*) was the most resistant Gram-negative bacteria with more than 20% resistance observed for amoxicillin (24.5%), ampicillin (23.5%), and trimethoprim/sulfamethoxazole (22.5%). Additionally, high resistance was observed for other first- and second-line antibiotics; for example, amoxicillin/clavulanic acid (13.2%), chloramphenicol (12.3%), and ciprofloxacin (8.2%), (Figure 1) [7]. Similarly for Gram-positive bacteria, of worrying concern is the high rate of resistance of *Streptococcus pneumoniae* to trimethoprim/sulfamethoxazole (64.3%) which is a commonly used antibiotic. Other commonly used antibiotics with a high percentage of resistance include oxacillin (32.2%), tetracycline (28.3%), penicillin (23.2%), amoxicillin (20.6%), and ampicillin (19.3%) (Figure 1) [7].

A proper laboratory-based surveillance program is necessary for identifying resistance and tracking its dissemination. Therefore, in order to extend the shelf life of antimicrobial agents in African countries, it is necessary to improve a number of factors, including access to diagnostic laboratories, surveillance of the emergence of resistance, regulation, and public, clinician/prescriber, and veterinary education regarding the proper use of antibiotics [4]. Implementing efficient and long-lasting AMR surveillance initiatives is difficult in low- and middle-income nations, such as those in Africa, in contrast to high-income nations. There is aa lack of institutional and infrastructure capabilities, a lack of funding and human resources, the underutilization of data already accessible, and limited transmission to regulatory agencies [7].

Poor AMR surveillance data over the entire continent of Africa make it difficult to comprehend the true scope and impact of AMR. When available, country data are not consistently gathered, shared with, or acknowledged by national entities, which restricts their power to influence national activities [11]. In response to this neglect, the 68th World Health Assembly (WHA) established a Global Action Plan (GAP) to address AMR with the overriding objective of raising awareness of AMR on a national and international level. The first two GAP-proposed goals, which center on raising public knowledge and comprehension of AMR through study and surveillance, are among a group of goals. Despite the GAP policy’s recommendation for the creation of national action plans (NAPs) and ongoing surveillance of priority pathogens, only two countries in the African region had adopted NAPs for AMR, and none had implemented any kind of national surveillance [6].

Antimicrobial resistance, which is presently thought to be responsible for more than 700,000 deaths annually worldwide, is causing increasing worry on a global scale. By 2050, if suitable steps are not taken to slow its spread, AMR will be responsible for nearly 10 million deaths and USD 100 trillion in annual costs [12]. Routine surveillance is therefore essential, especially in LMICs and Africa where AMR is expected to have the greatest impact [6]. As opposed to other health issues, AMR impacts every nation, regardless of its wealth or level of development, because resistant microbes know no national boundaries.

## 3. Quality Management

Effective disease prevention and management relies in accurate and time-efficient laboratory testing for patient care [13]. Healthcare systems are tasked with continuous quality improvement of patient care and safety [14]. High-quality microbiological diagnostics are essential for clinicians to make accurate diagnoses, formulate treatment plans, and subsequently monitor treatment effects on their patients [13,14,15,16,17]. Therefore, in developed countries laboratory diagnostics play a crucial role at every stage of disease management with clinicians relying on it for the diagnosis, monitoring, screening, and prognosis of disease. Here, information gathered through diagnostics, surveillance, monitoring, or follow-ups contributes to an understanding of the clinical picture, highlights opportunities for optimal patient care and improves the quality of care during the patient’s journey. This efficient use of laboratory test results in patient management results in the prudent use of antibiotics while reducing unnecessary treatments, improving diagnosis accuracy, monitoring disease progression, and evaluating treatment efficacy [18]. Miller et al. [19] proposed that the best outcomes for patients occur because of a strong partnership between the clinician and the laboratory. However, in Africa with scarce resources and skills required to perform accurate and reliable microbiology, most laboratories are often under-resourced, if they exist at all. Thus, with the nearly nonexistent quality assurance and control procedures, clinicians then resort to empirical antibiotics treatments or are reluctant to use laboratory tests in the management of patients, especially when results are discordant with clinical diagnosis [20]. In a cross-sectional study of 283 participants conducted in 2018 in Nigeria which assessed the reasons for poor utilization of laboratory testing in definitive diagnosis and infection management, several factors such as the physicians’ perception and attitude of the relevance of the laboratory as well as the apparent inadequacy of microbiology laboratory practices were proposed [21]. In a similar study, other challenges that affected the utilization of laboratory test results include delayed test results, inadequate test menus, a lack of feedback mechanisms, and poor quality assurance systems. In the author’s experience, even such basics as an adequate supply of the correct antibiotic discs for susceptibility can be a problem. Aid programs may supply antibiotics to clinicians but not think of the laboratory testing issues. Similarly, automated systems may be supplied in aid programs but there is no capacity for maintenance and repairs during break downs.

**Figure 2:**
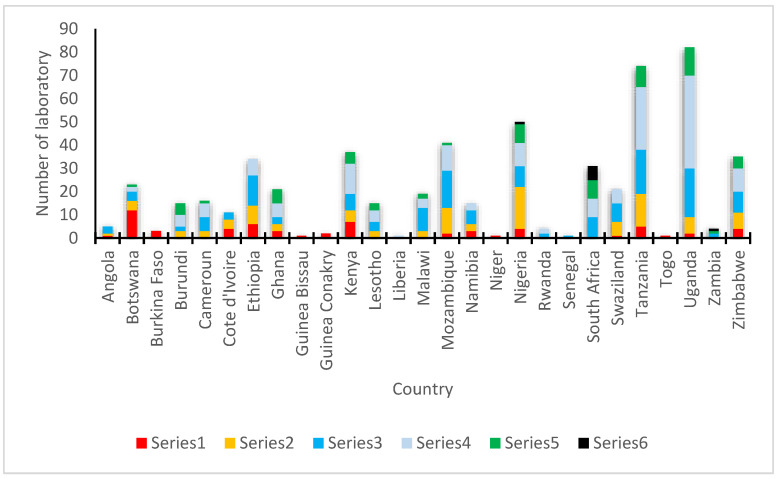
Star criteria used by SLIPTA to assess current level of quality and to identify areas for improvement for laboratories in Africa.

Laboratory quality assurance refers to the processes and procedures that laboratories use to ensure the accuracy and reliability of their test results. In Africa, laboratory quality assurance is an important tool for improving the quality of laboratory services and ensuring the reliability of test results. Despite the importance of laboratory quality assurance, many laboratories in Africa face significant challenges in implementing effective quality assurance programs. These challenges include a lack of resources, inadequate infrastructure, and limited access to training and technical support. To address these challenges, several organizations are working to support laboratories in Africa in their efforts to improve their quality assurance practices. One such organization is the African Society for Laboratory Medicine (ASLM), which promotes the use of the Stepwise Laboratory Quality Improvement Process Towards Accreditation (SLIPTA) framework [22]. SLIPTA provides a structured approach for laboratories to assess their current level of quality and identify areas for improvement using a star criteria (Figure 2). The framework includes a focus on quality assurance practices such as internal and external quality control, proficiency testing, and continuous quality improvement [22]. Countries are graded using the star criteria.

**Figure 3:**
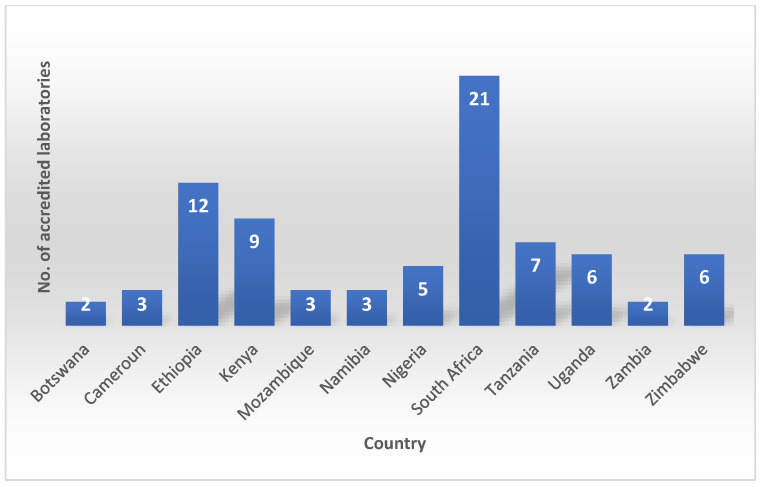
African laboratories achieving the International Organization for Standardization (ISO) 15,189 accreditation.

In addition to ASLM, other organizations such as the World Health Organization Regional Office for Africa (WHO/AFRO) and the US Centers for Disease Control and Prevention (CDC) are also working to support laboratory quality assurance in Africa. These organizations provide training, technical assistance, and other resources to help laboratories improve their quality assurance practices and achieve accreditation. Strengthening Laboratory Management Toward Accreditation (SLMTA) was developed as a task-based training and mentoring toolkit that provides laboratory personnel with the skills and knowledge they need to implement quality management systems [23]. SLMTA uses a multi-workshop implementation model to deliver training to laboratory managers. The training is composed of several tasks designed as tools and job aids which aid laboratory managers to enhance their management routines. Additionally, the program empowers managers with tools to initiate laboratory improvement measures with existing resources leading to better patient care and public health outcomes [23]. These programs have been successful in helping many laboratories improve their quality management systems with some African laboratories achieving the International Organization for Standardization (ISO) 15,189 accreditation (Figure 3).

Despite its successes, there are still challenges associated with its use in Africa. One challenge is the inherent lack of resources and infrastructure which limits the capacity and performance of laboratories and hinders their ability to comply with SLIPTA requirements. Additionally, poor communication, coordination, and integration among different levels of laboratory services can hinder the progress of these programs while insufficient political commitment by the local stakeholders, such as ministries of health, financial support, monitoring and evaluation, feedback mechanisms, and incentives for quality improvement result in a lack of sustainability. In addition to these challenges, there may also be cultural and organizational barriers to implementing SLIPTA. For example, some laboratory staff may be resistant to change or may not fully understand the importance of quality management systems. In addition, some laboratories may lack the leadership and support needed to successfully implement these programs. Finally, there is an urgent need for harmonization and alignment of SLIPTA with other existing or emerging laboratory quality initiatives in Africa, such as ASLM, SLMTA, and the International ISO 15189. This would help to avoid confusion, duplication, and contradiction among different standards, guidelines, and tools for laboratory quality improvement [24].

## 4. AMR Surveillance

Antimicrobial resistance surveillance has become an essential component of global health strategies to address AMR’s significant global threat. Surveillance plays a crucial role in guiding policies and infection prevention and control measures. Its significance lies in assessing the spread of AMR and monitoring the effectiveness of local, national, and global strategies. AMR surveillance involves monitoring trends and resistance patterns in different geographic regions, populations, and bacterial strains. This information is critical for identifying emerging resistance patterns, informing clinical practice and treatment guidelines, and guiding the development and deployment of new antimicrobial agents.

Several initiatives and programs have been established to strengthen AMR surveillance in Africa and globally, including the World Health Organization’s Global Antimicrobial Resistance and Use Surveillance System (GLASS) [25], the Africa Centres for Disease Control and Prevention (Africa CDC) Antimicrobial Resistance Surveillance Network (AMRSNET) [26], and the Fleming Fund, a UK government initiative aimed at tackling AMR in low- and middle-income countries [27]. These programs strengthen the national capacity to collect data on the prevalence and patterns of AMR from healthcare facilities, laboratories, and community settings, providing valuable insights into the current status of AMR and identifying areas where interventions are needed.

AMR surveillance in Africa faces several challenges, including limited laboratory capacity, inadequate data collection and analysis, and poor stakeholder coordination. However, with the growing recognition of AMR’s threat, there is increasing momentum to strengthen surveillance systems and improve data sharing and collaboration across the continent [28]. According to the Tripartite AMR Country Self-Assessment Survey (TrACSS) in 2022, only 16 of the 42 responding countries in the WHO African region use the relevant antimicrobial resistance surveillance data to inform operational decision-making and amend policies [29]. Furthermore, 70% have National Action Plans for AMR that are being implemented, and over half of them have already costed and budgeted their operational plans and adopted a monitoring mechanism [29].

One of the major obstacles is the insufficient demand for bacteriology diagnostics among healthcare providers because of their high costs and turnaround time. Moreover, health systems do not prioritize the supplies necessary for bacteriology diagnostics, which results in a consistent shortage of human resources and interruptions in the supply chain. Additionally, laboratory personnel may hesitate to conduct bacteriology diagnostics due to their time-consuming and inconvenient nature [30].

To ensure effective laboratory-based surveillance, it is essential to have a solid infectious disease diagnostic cycle, which involves clinicians sending samples for culture and sensitivity testing, a laboratory that can generate quality reports, a reporting system that delivers timely results to the clinician who requested the test, and a functional laboratory information system (LIS) that can provide information to the surveillance program. To have any functional surveillance system in the human health sector, it is necessary to establish at least one national reference laboratory in each country that can conduct phenotypic and genotypic evaluations of resistance and resistance genes [30]. Only Cabo Verde and São Tomé and Principe do not have a reference laboratory performing antimicrobial susceptibility testing [29].

### 4.1. Types of AMR Surveillance

For AMR surveillance, there are two approaches: active and passive surveillance. These methods are utilized to monitor and track the prevalence of AMR. Each method presents distinct advantages and disadvantages, and as such, a combination of both can offer a more comprehensive understanding of the dynamics of AMR [31].

Active surveillance is a method that involves proactively seeking out cases of AMR within a population. This can be achieved by systematically sampling patients or conducting focused surveillance among high-risk groups, such as hospital patients or individuals working with livestock. Active surveillance can provide a more nuanced comprehension of the prevalence of AMR and the emergence of resistant strains. A notable benefit of this approach is that it enables the collection of reliable data that can inform public health policies and guide the development of targeted interventions. Nonetheless, active surveillance can be resource-intensive and challenging to sustain over an extended period, particularly in resource-limited settings [32].

On the other hand, passive surveillance involves collecting data on AMR cases identified through routine clinical testing or other diagnostic procedures. This method relies on healthcare providers and laboratories to report cases of AMR to public health authorities. Passive surveillance can provide a more comprehensive view of a population’s AMR trends over time and help identify changes in resistance patterns. In contrast to active surveillance, passive surveillance is less resource-intensive and can be integrated into routine clinical practice. However, passive surveillance may be subject to underreporting, mainly if healthcare providers are not aware of the importance of reporting AMR cases or if reporting is not mandatory [33].

Active and passive surveillance have limitations, but when used together, they can provide a complete picture of the prevalence and dynamics of AMR in a population. This information can guide public health interventions, inform treatment decisions, and guide the development of new antibiotics and other therapies. It is essential to distinguish between the terms “active” and “passive” in antimicrobial resistance (AMR) surveillance, as they are often used inaccurately and inconsistently. For instance, performing whole-genome sequencing on routine isolates is considered passive surveillance, despite active testing being involved. Genuine active surveillance programs are rare, whereas there is a wide range of passive surveillance programs, including routine data collection or gathering additional patient information, conducting further tests on isolates, and implementing new alerts and actions. Most national AMR surveillance programs describe themselves as “active” even though they still rely on routine clinical samples, rendering them not entirely active according to traditional definitions.

In the WHO African region, among the 42 countries that participated in the TrACSS survey in 2022 [29], 14 countries possess a standardized national system for monitoring antimicrobial resistance (AMR). These countries collect data on common bacterial infections in both hospitalized and community patients. They have also established a network of surveillance sites, a designated national reference laboratory for AMR, and a national coordinating center responsible for generating reports on AMR. On the other hand, 20 countries compile their data at the local level, while 4 countries gather their data nationally. However, these countries lack a standardized approach, national coordination, and/or quality management. Nonetheless, Lesotho, Central African Republic, and South Sudan face challenges in generating data on AMR.

### 4.2. Genomic Surveillance

Genomic surveillance of AMR entails the use of state-of-the-art sequencing technologies to investigate the genomic evolution and dissemination of antibiotic-resistant bacteria. This approach involves the meticulous analysis of bacterial strains’ genetic material to pinpoint specific resistance mechanisms and mutations responsible for AMR. Given its capacity to promptly identify newly emerging resistant strains and facilitate the development of more precise treatment and preventive measures, this approach is gaining prominence in the fight against AMR.

Genomic surveillance of AMR utilizes multiple techniques, with whole-genome sequencing (WGS) being a prevalent method. WGS entails sequencing the complete genome of a bacterial strain to pinpoint genetic mutations linked to AMR. This technique yields comprehensive data on the genetic mechanisms underlying resistance and enables the detection of new or developing strains of resistant bacteria [34]. Another approach is metagenomics, where all genetic material in a given sample, including bacterial DNA, is sequenced. This technique can effectively detect resistant bacteria in intricate settings such as hospitals or farms and furnish valuable information on the dissemination of resistant strains within these environments [35].

Genomic surveillance has several benefits over traditional surveillance methods, such as culturing bacteria on agar plates, which can be time-consuming and may miss essential strains. Genomic surveillance is also more sensitive and specific, allowing for detecting resistance mechanisms that may not be visible with traditional methods [36].

Genomic surveillance is gaining traction in Africa as a tool for preventing and controlling emerging infections, as seen in the 2018 outbreak of Lassa fever virus in Nigeria and Ebola virus outbreaks in the Democratic Republic of the Congo (2018–2020) [37,38]. Genomic data were used with other data to determine transmission dynamics, assess spatiotemporal aspects of the epidemic, and evaluate the efficacy of diagnostic tests and vaccines [39]. Thus, the capacities exist on the content and can be repurposed for antimicrobial resistance.

The data generated through genomic surveillance can inform public health policy and guide the development of new antibiotics and other treatments for resistant infections. For example, the data can help identify high-risk populations, such as patients in hospitals or individuals who work with livestock, and inform targeted intervention strategies [39].

### 4.3. The Need for a One-Health Surveillance System

One Health is gaining attention in Africa for AMR surveillance due to the high burden of infectious diseases, poor health infrastructure, and weak regulatory systems. One Health aims to ensure the wellbeing of humans, animals, and the environment. It is a comprehensive strategy that emphasizes prevention, prediction, detection, and response to prevent global health crises such as the COVID-19 pandemic. This approach requires collaboration across various sectors and communities to address the underlying causes of health issues and develop sustainable solutions. One Health is relevant to public health, veterinary medicine, environmental protection, and other sectors. It is particularly applicable to ensuring food and water safety, promoting proper nutrition, controlling animal-to-human diseases, managing pollution, and addressing antibiotic-resistant microbes. According to TrACSS 2022, only 19 countries have established or started implementing an integrated multi-sectoral surveillance system for antimicrobial resistance [29].

The African Union Inter-African Bureau for Animal Resources (AU-IBAR) has established the One Health Coordination Group on Zoonotic Diseases program and developed a framework for One Health practice based on collaboration between various stakeholders. The framework emphasizes the importance of collecting and sharing data on AMR in humans, animals, and the environment. A One Health approach to AMR surveillance in Africa is critical for addressing the increasing threat of drug-resistant infections in both humans and animals. Additionally, to support these integration efforts, SILAB for Africa (SILABFA), a surveillance system widely used for laboratory diagnostic activities in animal sectors, is now working on the integration of the human data, specifically through the integration and enhancing the interoperability with WHONET, a software widely used for AMR surveillance in human laboratories [40].

Effective implementation of One Health surveillance strategies for antimicrobial resistance (AMR) in Africa is hindered by limited inter-sectoral collaboration, communication, and coordination among the animal health, environmental health, and human health sectors. Silos and insufficient data sharing due to issues of ownership and confidentiality also pose a significant challenge to One Health surveillance.

Currently, after excluding the four countries with no NAPs, 32 out of the 38 countries developing or implementing their NAPs are ensuring an integrated AMR national planning process with other existing action plans and/or strategies, according to TrACSS 2022, and 23 countries are already implementing AMR awareness-raising activities with other sectors and stakeholders [29].

## 5. Future Directions

Considerable progress has been made globally to increase the knowledge and under-standing of AMR especially in LMIC using effective communication, education, and training. However, due to the high burden of infectious diseases in African countries and the need to lower the incidence of illness through good sanitation, hygiene, and infection prevention measures, African countries should work on:

- Using a continent-wide approach to identify existing gaps in the current regulatory framework for diagnostic tests.
- Promote adoption and enforcement on governance regulations for laboratories in the continent.
- Support the harmonization of a country/continent-led external quality assurance scheme for laboratory management.
- Identify barriers mitigating AMR surveillance and develop pathways of addressing barriers politically, financially, and technically.
- Harness genomic sequencing expertise developed during the COVID-19 pandemic to create a cost-effective snap-shot surveillance system to inform public health approaches.
- Improve the availability of continent-wide AMR data to better understand the prevalence of antimicrobial resistance across human health and animals using the One-Health surveillance approach.

## 6. Limitation

Some information used for this review was retrieved from TrACSS. The TrACSS assessment used for the AMR surveillance information has several limitations. Firstly, self-reporting bias can arise as countries voluntarily provide data per their assessment. Secondly, inaccurate or incomplete data have been reflected due to incomplete submissions or no submissions by some countries in the region for various reasons, resulting in data gaps. Thirdly, interpretation variability and validity among countries can cause inconsistent responses, complicating data comparison. Therefore, validating the accuracy and reliability of reported data becomes challenging without independent verification. Finally, since the data provided are not associated with any scoring system, it is difficult to interpret the data for comparability between the countries.

## 7. Conclusions

In conclusion, understanding the full extent and impact of AMR in Africa will not be possible unless we have reliable AMR surveillance and laboratory quality assurance. When available, country data are not consistently gathered, shared, or acknowledged by national entities, which restricts their capacity to have an impact on national actions. Laboratory diagnostics should play a crucial role at every stage of disease management with clinicians relying on it for the diagnosis, monitoring, screening, and prognosis of disease.

Laboratory quality assurance is an important tool for improving the quality of laboratory services and ensuring the reliability of test results. Despite the importance of laboratory quality assurance, many laboratories in Africa face significant challenges in implementing effective quality assurance programs.

AMR surveillance in Africa faces several challenges, including limited laboratory capacity, inadequate data collection and analysis, and poor stakeholder coordination. However, with the growing recognition of AMR’s threat, there is increasing momentum to strengthen surveillance systems and improve data sharing and collaboration across the continent which will eventually lead to better control of AMR.

## Data Availability

No new data were created or analyzed in this study. Data sharing is not applicable to this article.
